# Pandemic (H1N1) 2009 Surveillance for Severe Illness and Response, New York, New York, USA, April–July 2009

**DOI:** 10.3201/eid1608.091847

**Published:** 2010-08

**Authors:** Sharon Balter, Leena S. Gupta, Sungwoo Lim, Jie Fu, Sharon E. Perlman, for the New York City 2009 H1N1 Flu Investigation Team

**Affiliations:** New York City Department of Health and Mental Hygiene, New York, New York, USA

**Keywords:** Pandemic (H1N1) 2009, H1N1, nH1N1, New York, surveillance, hospitalization, risk factors, influenza, viruses, poverty, research

## Abstract

On April 23, 2009, the New York City Department of Health and Mental Hygiene (DOHMH) was notified of a school outbreak of respiratory illness; 2 days later the infection was identified as pandemic (H1N1) 2009. This was the first major outbreak of the illness in the United States. To guide decisions on the public health response, the DOHMH used active hospital-based surveillance and then enhanced passive reporting to collect data on demographics, risk conditions, and clinical severity. This surveillance identified 996 hospitalized patients with confirmed or probable pandemic (H1N1) 2009 virus infection from April 24 to July 7; fifty percent lived in high-poverty neighborhoods. Nearly half were <18 years of age. Surveillance data were critical in guiding the DOHMH response. The DOHMH experience during this outbreak illustrates the need for the capacity to rapidly expand and modify surveillance to adapt to changing conditions.

## Abstract 2

Health departments need the capacity to rapidly expand and modify surveillance during a changing outbreak.

On April 23, 2009, a nurse from a high school in New York City (NYC) called the Department of Health and Mental Hygiene (DOHMH) to report an outbreak of respiratory illness (*1*). The cause of the outbreak was rapidly confirmed to be influenza A pandemic (H1N1) 2009 virus. This outbreak was detected just a few days after initial reports of mild disease caused by pandemic (H1N1) 2009 virus in California and Texas (*2**,**3*) and at the same time as an outbreak of severe respiratory disease associated with pandemic (H1N1) 2009 virus in Mexico (*4*). Information about the clinical severity and transmission characteristics of this new influenza virus was limited. Given preliminary media reports about the Mexican outbreak and concern that NYC might also experience widespread severe disease, DOHMH launched a large-scale public health response.

Before the spring of 2009, DOHMH routine surveillance systems for influenza included 1) syndromic surveillance for medication sales, school absenteeism, and emergency department visits for influenza-like illness (ILI) (*5**,**6*); 2) electronic laboratory reporting of confirmed cases from commercial and hospital laboratories; 3) active surveillance of all NYC virology laboratories to determine the weekly number of specimens submitted for influenza testing and the percentage of those positive; 4) typing samples of influenza isolates obtained from patients in NYC hospitals at the DOHMH Public Health Laboratory (PHL); 5) enhanced passive surveillance for pediatric influenza deaths; 6) monitoring trends in influenza and pneumonia-related mortality through the DOHMH Vital Registry; and 7) monitoring outpatient ILI through the Centers for Disease Control and Prevention (CDC; Atlanta, GA, USA) Influenza-like Illness Surveillance Network (*7*), a sentinel network through which providers reported weekly on the proportion of ILI in their practices during influenza season. The DOHMH had also created a plan for local response to a potential influenza pandemic, including enhanced surveillance to guide public health officials in determining how to prioritize use of antiviral agents and vaccines (*8*). Surveillance data could also inform community control measures, such as school closures. Proposed surveillance strategies in this plan focused on mechanisms for monitoring trends in hospitalizations and deaths, but not necessarily for trying to count every severe case. Methods were also proposed for obtaining more detailed clinical and epidemiologic data for a sample of cases.

The DOHMH also has an incident command system (ICS), an agency-wide structure for addressing and responding to emergencies that is different from the usual DOHMH structure. Divided into 10 sections, the ICS is led by an incident commander who reports directly to the Commissioner of Health (*9*). All DOHMH employees are assigned to a section within the ICS and can be called on to assist their section upon activation of the system. In a public health emergency, the Surveillance and Epidemiology Section establishes and conducts surveillance to assess the illness and deaths associated with the event and conducts any needed epidemiologic studies to guide the public health response. ICS activation provides surge capacity by increasing the workforce available to conduct surveillance or epidemiologic activities beyond the staff members who are normally responsible for the specific disease or public health issues involved in the emergency.

We describe some of the surveillance methods used in the investigation of pandemic (H1N1) 2009 in NYC from April to July 2009. DOHMH investigated the high school outbreak (*1**,**10*), and set up an enhanced citywide surveillance system to track the scope and severity of infections. The agency also prioritized identification and diagnostic testing of patients with severe or fatal cases of ILI in hospitals or clusters of those with ILI in schools and other congregate settings; this surveillance was essential because evidence of severe pandemic (H1N1) 2009 would have prompted more aggressive public health control measures. In addition, because surveillance of cases in hospitalized patients, and particularly of fatal cases, was an important part of this investigation, we provide an overview of epidemiologic findings among hospitalized patients.

## Methods

On Saturday, April 25, 2009, when preliminary laboratory results suggested a likely pandemic (H1N1) 2009 outbreak at high school A, the DOHMH activated its ICS and initially mobilized >200 staff members for a large-scale public health response (later adding additional staff). From April 25 through May 8, the agency also expanded its hours of operation to 7 days a week from 9:00 am to 9:00 pm; staff worked in shifts to cover the extended hours.

The ICS was deactivated on May 8, since minimal evidence existed of community circulation of pandemic (H1N1) 2009. By mid-May, however, DOHMH noticed an increase in ILI, especially in schoolchildren. On May 17, 2009, the first NYC death from pandemic (H1N1) 2009 virus occurred. In response to these developments and to increasing reports of hospitalized case-patients, DOHMH reactivated its ICS on May 19. This second activation continued until July 7, 2009.

### Enhanced Citywide Surveillance

#### Active Surveillance for Critically Ill Case-Patients

Starting April 26, the DOHMH conducted active citywide surveillance in hospital intensive care units (ICUs) for severe, unexplained, febrile respiratory illnesses (defined as a temperature >100.4°F (>38°C) and pneumonia, acute respiratory distress syndrome, or respiratory distress (as diagnosed by clinicians) with no known cause. DOHMH staff contacted all 57 NYC hospitals with medical or pediatric ICUs daily by telephone and queried the clinician in charge of the ICU that day to determine the number of patients with conditions that met the surveillance definition. Active ICU surveillance was discontinued on May 8 since few cases of severe illness were being identified**.**

#### Enhanced Passive Surveillance for Hospitalized Case-Patients with Noncritical Illness

To ascertain the number of hospitalized patients with pandemic (H1N1) 2009 outside of the ICU setting, DOHMH relied on enhanced passive surveillance. Providers were notified of reporting requirements through the NYC Health Alert Network, which sends faxes and email alerts to 29,000 clinicians and healthcare institutions in NYC, and through daily conference calls with all NYC acute care facilities. DOHMH set up a dedicated NYC telephone access line to triage provider calls. Providers were initially asked to report any hospitalized patients outside of the ICU setting with severe, unexplained, febrile respiratory illnesses (as defined above). However, because of the increasing number of calls and limited staff and laboratory testing capacity at the NYC PHL, beginning on May 12, providers were asked to only report non-ICU cases of severe, febrile respiratory illness if initial test results were positive for influenza A virus by enzyme immunoassay, PCR, direct fluorescent antibody test, or virus culture at the hospital laboratory. However, DOHMH continued to accept reports on all patients with febrile respiratory illness who were in the ICU or were receiving ventilation, regardless of influenza testing status.

#### Active Laboratory Surveillance

During the week after the recognition of pandemic (H1N1) 2009 in NYC (April 25–30), DOHMH actively collected specimens from laboratories chosen to be geographically representative of the city to determine whether evidence existed of community circulation of the pandemic virus that was not associated with the outbreak at the high school. Five sentinel laboratories were selected and asked to submit 1–3 influenza A virus–positive specimens from the previous 2 days to the NYC PHL to test for pandemic (H1N1) 2009 virus.

#### Case-Patient Interviews

During the first 3 weeks of the outbreak, DOHMH staff attempted telephone interviews of all patients (or their proxies) who had confirmed pandemic (H1N1) 2009 for demographic, epidemiologic, and clinical information. Providers of care for hospitalized case-patients were also interviewed to gather information about patient demographics, underlying conditions, and clinical course of illness. Once community circulation in NYC was established, DOHMH stopped interviewing patients about possible risk of exposure (e.g., travel to Mexico, school attendance).

### Surveillance for Deaths

To track pandemic (H1N1) 2009–related deaths, DOHMH asked that hospitals report any fatal cases of unexplained, acute, febrile respiratory illness to DOHMH and to the Office of the Chief Medical Examiner (OCME). The OCME collected specimens and performed autopsies on any patient whose death was preceded by a sudden, unexplained, febrile respiratory illness, as well as for all pediatric patients who died with clinically compatible illness in which there was a positive influenza test result, a sudden unexplained death thought to be due to a natural cause, or death of a child from an unknown febrile respiratory illness. If no testing results for pandemic (H1N1) 2009 virus were available from the hospital, OCME collected a postmortem nasopharyngeal swab specimen for influenza diagnostic testing at PHL. In addition, the dataset of patients who tested positive for pandemic (H1N1) 2009 virus was matched weekly with the NYC Vital Records database of recent deaths in NYC to ensure that no pandemic (H1N1) 2009 deaths were missed.

### Laboratory Methods

DOHMH physicians screened reported potential cases to determine if they met testing criteria, and if so, nasopharyngeal specimens were requested. Recognizing the need to prioritize PHL resources for hospitalized patients and those with fatal cases, DOHMH specifically requested that clinicians not test patients with mild ILI unless the patient was part of a reported cluster in a school, jail, nursing home, or other congregate setting.

Specimens were initially tested for influenza A or B viruses, and then, if positive for influenza A, were further tested for seasonal influenza A virus (H1N1 or H3N1) by using the QIAamp Viral RNA manual extraction method (QIAGEN, Valencia, CA, USA) and real-time reverse transcription–PCR by using the Cepheid SmartCycler (Cepheid, Sunnyvale, CA, USA). Initially, specimens that were positive for influenza A virus, but not seasonal influenza A virus subtypes H1N1 or H3N1, and suspected to be pandemic (H1N1) 2009 virus were sent to CDC for confirmation. Then, on May 11, the PHL started to perform the CDC Influenza Virus Real-time reverse transcription–PCR detection and characterization panel for pandemic (H1N1) 2009 virus on all nonseasonal influenza A specimens by using a high-throughput system including an automated extraction system and ABI7500 Fast-Dx (Life Technologies, Carlsbad, CA, USA). Beginning on May 20, PHL performed the same CDC assay on all influenza specimens by using the same high throughput system.

A confirmed case of pandemic (H1N1) 2009 was defined as a person who had a specimen that was PCR positive for pandemic (H1N1) 2009 virus. A probable case was defined as a patient with nonsubtypeable influenza A virus infection for whom confirmatory testing was not conducted. Confirmatory influenza testing was performed at PHL, CDC, or the New York State Wadsworth Center Laboratory.

### Analytic Methods

We analyzed surveillance data to describe NYC residents who were hospitalized with pandemic (H1N1) 2009 in NYC from the start of the first ICS activation to the end of the second activation (April 24–July 7). We also calculated pandemic (H1N1) 2009 rates by dividing the number of confirmed and probable cases among hospitalized patients by NYC population counts from the US Census 2000. We examined rates by demographic characteristics of hospitalized patients and performed direct age-adjustment by using weights based on US Census 2000 (*11*).

Additionally, patient poverty level was assessed by linking ZIP code of residence with income and population data from the US Census 2000. We defined neighborhoods using the United Hospital Fund (UHF) designation, which aggregates adjoining ZIP codes to create 42 NYC neighborhoods (*12*). We then created a neighborhood poverty variable by categorizing UHF neighborhoods into tertiles (low-, medium-, and high-poverty neighborhoods) based on the percentage of residents living <200% of the federal poverty level, according to the US Census 2000, and calculated rates for confirmed and probable pandemic (H1N1) 2009 cases by UHF neighborhood poverty status (*11*). Poverty data were available for 993 of the 996 persons who were hospitalized with confirmed or probable pandemic (H1N1) 2009.

For all analyses, significance was determined at p<0.05. All statistical analyses were conducted by using SAS 9.2 (SAS Institute Inc., Cary, NC, USA).

## Results

### Hospitalizations

During April 24–May 7, corresponding to the first ICS activation, 15 patients with confirmed or probable cases of pandemic (H1N1) 2009 were hospitalized (median stay was 1 day). At that time, most cases were linked to the high school influenza A outbreak and only 2 case-patients reported travel to Mexico. No deaths had been reported. Since few cases of severe illness had occurred, the ICS was deactivated, and staff who would normally be involved in communicable disease outbreak investigations continued to monitor pandemic (H1N1) 2009 activity.

**Figure 1:**
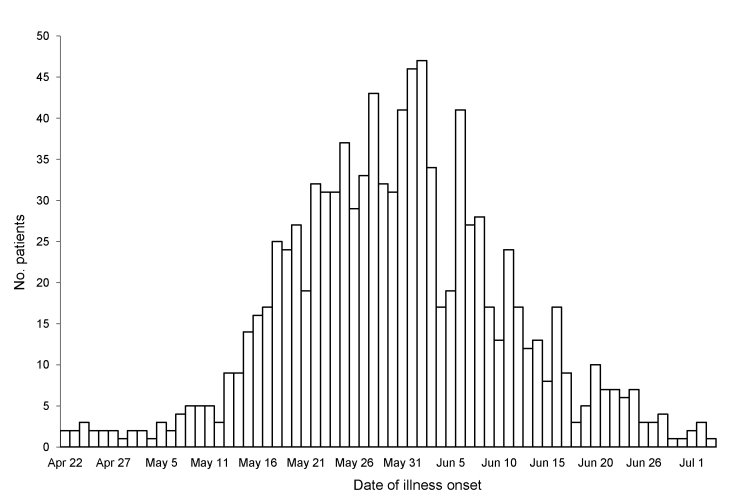
Hospitalized patients with confirmed or probable pandemic (H1N1) 2009, by date of onset, New York, New York, USA, April 24–July 7, 2009. Onset date was missing for 98 patients with confirmed pandemic (H1N1) 2009 and 16 with probable pandemic (H1N1) 2009. Surveillance data as of August 25, 2009.

**Figure 2:**
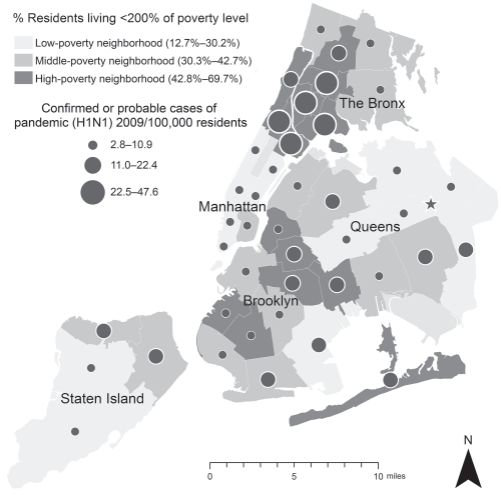
Age-adjusted rates of hospitalization for confirmed or probable pandemic (H1N1) 2009, by neighborhood poverty level, New York, New York, USA, April 24–July 7, 2009. Direct age standardization was performed by using weights from the 2000 US Census (*11*). Of 996 total patients, 993 had complete poverty data available. Star represents location of high school A.

By July 7, the end of the second ICS activation, 996 patients had been hospitalized. The distribution of 996 hospitalized case-patients (929 confirmed and 67 probable) over time, including the increased incidence in late May, can be seen in Figure 1. From April 24 through July 7, the estimated age-adjusted rate of confirmed and probable pandemic (H1N1) 2009 hospitalizations was 12.3/100,000 NYC residents (95% confidence interval [CI] 11.8–13.4). The rate among patients <4 years of age (40.9/100,000, 95% CI 35.6-46.3) was almost 7× that among those >65 years of age (6.0/100,000, 95% CI 4.5–7.7) (Table). The estimated age-adjusted rate of pandemic (H1N1) 2009 hospitalized patients in high-poverty neighborhoods (18.4/100,000, 95% CI 16.8–20.1) was significantly higher than that in low-poverty neighborhoods (8.9/100,000, 95% CI 7.6–10.4) (Table; Figure 2).

**Table:** Demographic characteristics of patients hospitalized with confirmed or probable pandemic (H1N1) 2009, New York, NY, USA, April 24–July 7, 2009*

Characteristic	No. (%) patients with confirmed or probable pandemic (H1N1) 2009	% All residents of city†	Crude rate/100,000 residents (95% CI)	Age-adjusted rate/ 100,000 residents (95% CI)‡
Total	996		12.3 (11.5–13.1)	12.6 (11.8-13.4)
Age group, y				
0–4	224 (22)	6.7	40.9 (35.6–46.3)	–
<2	128 (13)	2.7	58.1 (48.0–68.1)	–
2–4	96 (10)	4.0	29.4 (23.8–35.9)	–
5–24	297 (30)	27.4	13.3 (11.8–14.8)	–
5–17	197 (20)	17.4	13.9 (12.0–15.9)	–
18-24	100 (10)	10.0	12.3 (9.9–14.7)	–
25–64	419 (42)	54.0	9.6 (8.6–10.5)	–
25–49	245 (25)	39.5	7.6 (6.7–8.6)	–
50–64	174 (17)	14.5	14.8 (12.6–16.9)	–
>65	56 (6)	14.5	6.0 (4.5–7.7)	–
Sex§				
M	468 (47)	47.4	12.2 (11.1–13.3)	12.5 (11.4–13.7)
F	526 (53)	52.6	12.3 (11.3–13.4)	12.9 (11.9–14.1)
Poverty status§¶				
High-poverty area	498 (50)	32.7	18.8 (17.1–20.4)	18.4 (16.8–20.1)
Medium-poverty area	323 (33)	40.9	9.7 (8.7–10.8)	10.1 (9.1–11.3)
Low-poverty area	172 (17)	26.4	8.0 (6.8–9.2)	8.9 (7.6­–10.4)

*CI, confidence interval. †Percentage of all residents of the city in each category. Estimates based on 2000 US Census data (*11*).  ‡Direct age standardization was performed by using weights based on 2000 US Census data (*11*). Median age for hospitalized patients was 23 y and for all city residents was 34 y. §2 persons were missing sex data; 3 persons were missing poverty data. ¶Neighborhood poverty was based on the tertiles of percentage of residents living <200% of the federal poverty level according to the 2000 US Census (*11*).

### Deaths

The first NYC death occurred on May 17. Additional information about NYC pandemic (H1N1) 2009 deaths has been published elsewhere (*13*).

## Discussion

The experience of NYC with pandemic (H1N1) 2009 demonstrates the need for flexibility in surveillance approaches and ongoing modification of surveillance methods to best respond to a changing public health emergency. Although DOHMH had not planned to do such intensive active and enhanced surveillance during an influenza pandemic (*9*), active case-based surveillance was initially implemented because little was known about the severity of this novel strain of H1N1, and public health officials were concerned on the basis of initial media reports from Mexico. To learn more about the severity of illness, DOHMH focused on surveillance of hospitalized cases and deaths. Surveillance and reporting requirements were modified when it became clear that circulation of pandemic (H1N1) 2009 was citywide, but surveillance for deaths and hospitalized cases continued to help officials assess the severity and at-risk groups for pandemic (H1N1) 2009, and the resulting information helped inform DOHMH planning and response to this new virus.

Approximately half of hospitalized patients lived in a high-poverty neighborhood; this association between poverty and severe illness has been reported for seasonal influenza (*14*). Our finding of the association between young age and severe illness is consistent with other studies (*15**–**20*), and the greater proportion of persons 0–17 years of age in low-income neighborhoods in NYC (*21*) may contribute to this distribution. Other possible explanations include higher attack rates among residents living in crowded housing, or that residents with known risk conditions in high-poverty neighborhoods may be less likely to receive early treatment or prophylaxis, given that the proportion of people without personal doctors is higher in high-poverty areas relative to low-poverty areas (20% vs. 11%) (*21*). In addition, the proportion of uninsured persons in low-income areas (18%) is higher than the proportion in high-income areas (9%), according to NYC’s Community Health Survey from 2008 (*21*). Future studies should assess poverty status and its relationship to severe influenza illness.

Our analysis had several limitations. By limiting testing to those patients who had positive influenza A test results (unless patients were in the ICU), our surveillance approach systematically undercounted hospitalized patients with pandemic (H1N1) 2009. Although this enabled us to monitor hospitalization trends, we most likely do not have a complete count of cases. Published studies have found a wide range of results for the sensitivity of rapid influenza testing for the pandemic (H1N1) 2009 strain (17%–70%) (*22*–*25*). Applying the published range of sensitivities to our results would suggest that the true number of hospitalized patients in NYC ranged from 1,400 to 6,000, which is 1.5–7.0× higher than those for cases detected and confirmed. Also, because of the limited amount of data collected on all patients, we were unable to examine variables at the individual level; such data (for example, having a primary care physician and insurance status) may have modified the findings regarding the relationship between poverty and severe illness. Lastly, demographic and economic information was from 2000, and changes may have occurred.

Surveillance data from the spring outbreak informed NYC planning and response to pandemic (H1N1) 2009 during the 2009–10 fall and winter influenza season. Because young children represented a large proportion of hospitalized cases and because of the role children likely play in transmission, NYC created a school-based vaccination program for elementary and middle schoolchildren and vaccinated all children who had parental consent. In the fall of 2009, >60 influenza diagnostic and treatment community based centers were established for persons with ILI who did not have a primary care physician; an advice hotline, staffed by nurses, was created to answer questions and help connect NYC residents to care. Antiviral medications were made available to those who could not afford them, and points of distribution provided the vaccine free of charge to New Yorkers, initially targeting those who had risk factors for severe pandemic (H1N1) 2009 as identified in NYC and elsewhere (*15**–**20**,**26**,**27*).

DOHMH has continued to use emergency room and outpatient syndromic surveillance systems to follow trends in influenza-like activity citywide. We also requested passive reporting of influenza hospitalizations by all city hospitals and collected some data on clinical status and risk factors. Finally, to more effectively monitor the clinical and epidemiologic characteristic of pandemic (H1N1) 2009 during the fall and winter seasons, we established a sentinel hospital surveillance program at 5 sites where active surveillance and influenza testing were conducted on any patient with fever and respiratory syndromes. Collection of isolates from sentinel hospitals and active laboratory surveillance also allowed circulating influenza subtypes, as well as antiviral resistance, to be monitored. Surveillance guided and informed the NYC response to pandemic (H1N1) 2009, and this experience will help NYC plan a response to future epidemics.
